# Research progress of intestinal microbiota on cognitive dysfunction after spinal cord injury

**DOI:** 10.1016/j.isci.2025.113554

**Published:** 2025-09-12

**Authors:** Chunping Tian, Xiaowei Chang, Jiajun Wu, Linfeng Xiao, Jiani Du, Qianqian Hu, Yanling Yang

**Affiliations:** 1 Yan’an Medical College of Yan’an University, Yan’an, Shaanxi 716000, China

**Keywords:** Cognitive neuroscience, Microbiome

## Abstract

Alterations in the gut microbiota are among the most common phenomena following spinal cord injury, often accompanied by cognitive impairment. Numerous studies have reported intrinsic links among these three aspects. However, the molecular mechanisms by which the gut microbiota influence cognitive function after spinal cord injury, as well as strategies for modulating the gut microbiota to treat cognitive deficits, remain inadequately summarized. Therefore, this review provides a comprehensive summary of the key mechanisms by which gut dysbiosis contributes to cognitive impairment and discusses therapeutic strategies targeting the gut microbiota. These insights may offer a theoretical basis for further research and microbiota-based drug development for cognitive dysfunction following spinal cord injury.

## Graphical abstract

**Figure:**
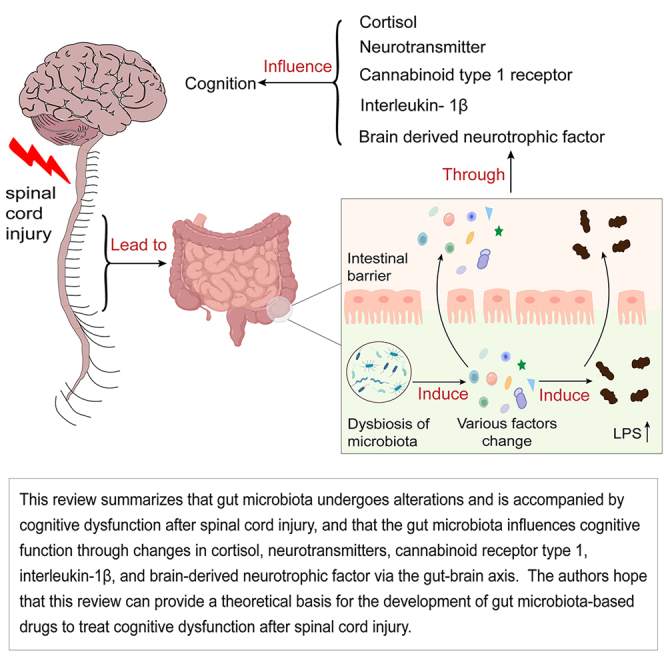

## Introduction

Spinal cord injury (SCI) is a severe traumatic disorder of the central nervous system that not only results in the loss of motor and sensory functions but also frequently leads to a range of complex complications, including impairments in attention, executive function, and memory.^1^^,^^2^^,^^3^ Numerous studies have shown that individuals with SCI have a 13-fold higher risk of developing cognitive impairment compared to the general population,^4^imposing a significant burden on patients, families, and healthcare providers.^5^ Traditionally, cognitive dysfunction after SCI has been attributed primarily to direct neural damage and secondary neuroinflammatory responses.^6^ However, with the advancement of research, the gut microbiota has emerged as a promising and increasingly recognized area of investigation.

The gut microbiota refers to a diverse community of microorganisms residing in the gastrointestinal tract, maintaining a close symbiotic relationship with the host. The gut-brain axis generally refers to the bidirectional communication between the central nervous system (CNS) and the enteric nervous system. It can influence the normal development of the CNS and the pathogenesis of related diseases through multiple pathways, such as regulating intestinal barrier permeability, the hypothalamic-pituitary-adrenal (HPA) axis, immune stress, and neurotransmitter synthesis.^7^^,^^8^^,^^9^ In healthy individuals, the gut microbiota maintains a state of dynamic equilibrium. However, gastrointestinal dysfunction resulting from SCI can disrupt this balance, leading to gut dysbiosis.^10^ Alterations in the gut microbiota can compromise intestinal barrier integrity and modulate brain function via the bidirectional microbiota–gut–brain axis,^11^thereby contributing to cognitive dysfunction.^12^ These findings suggest that the gut microbiota may play a role in SCI-associated cognitive impairment.

**Figure 1:**
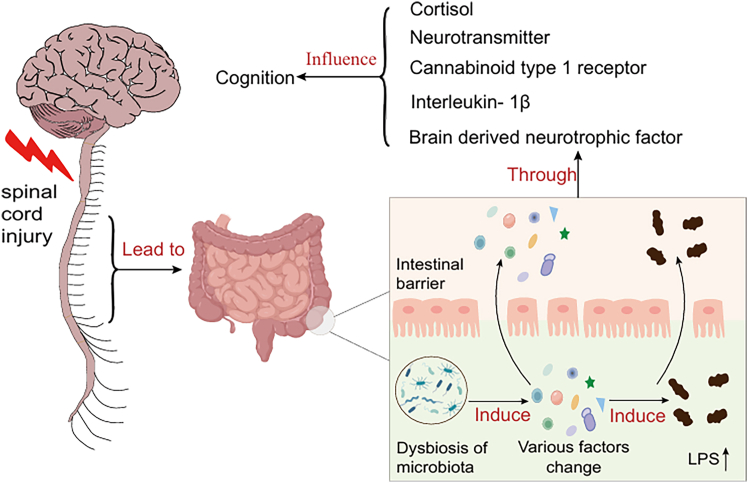
The dysbiosis of gut microbiota after spinal cord injury affects cognitive function Spinal cord injury can lead to changes in the gut microbiota and various factors, which increase the level of lipopolysaccharides. These factors and lipopolysaccharides enter the damaged intestinal barrier and then affect cognitive function by influencing the changes in cortisol, neurotransmitters, cannabinoid receptor type 1, interleukin-1β, and brain-derived neurotrophic factor.

Existing literature indicates a close relationship between gut microbiota and cognitive dysfunction following SCI;^13^^,^^14^however, the specific mechanisms by which gut microbiota contribute to this impairment remain poorly defined. Therefore, this review first outlines the interrelationships among gut microbiota, SCI, and cognitive dysfunction, and then systematically summarizes how alterations in gut microbiota may influence post-SCI cognitive function through changes in cortisol, neurotransmitters, cannabinoid type 1 receptor (CB1), interleukin-1β (IL-1β), and brain-derived neurotrophic factor (BDNF) (Figure 1). Based on these mechanisms, potential therapeutic strategies targeting the gut microbiota to alleviate cognitive impairment after SCI are discussed. Finally, the review provides an outlook on future research directions concerning gut microbiota, SCI, and cognitive dysfunction, offering valuable insights for the prevention and mitigation of cognitive deficits following SCI.

## The relationship between gut microbiota, spinal cord injury, and cognitive dysfunction

### Gut microbiota and spinal cord injury

The gut microbiota refers to a complex community of microorganisms residing in the gastrointestinal tract, comprising bacteria, fungi, viruses, and other microbial species. Based on their functional roles, gut microbes can be broadly classified into three categories: beneficial, harmful, and neutral bacteria. Beneficial bacteria are primarily anaerobes, such as *Lactobacillus*, *Bifidobacterium*, and *Actinomycetes*, which enhance host immunity, promote intestinal peristalsis, and aid in digestion and nutrient absorption, thereby contributing to overall health. Harmful bacteria include *Proteus*, *Bacteroides*, *Escherichia coli*, and *Enterococcus*, which, in excessive amounts, can produce bacterial toxins detrimental to host health. Neutral bacteria, which lie between beneficial and harmful species, may shift in function depending on the dominance of either side, thereby influencing host health.^15^ At the phylum level, the gut microbiota primarily comprises four major groups: *Firmicutes*, *Bacteroidetes*, *Actinobacteria*, and *Proteobacteria*.^16^ The first three are generally considered beneficial, while *Proteobacteria* are typically classified as harmful. Under normal conditions, these microbial communities exist in a relatively stable equilibrium, collaboratively maintaining intestinal mucosal barrier integrity and immune homeostasis. This balance prevents harmful substances from entering the bloodstream and triggering excessive inflammation, thereby supporting overall health. However, the disruption of this balance—gut dysbiosis—has been associated with various diseases, including Parkinson’s disease (PD),^17^ Alzheimer’s disease (AD),^18^ and depression.^19^

Studies have shown that SCI compromises the integrity of the blood–spinal cord barrier, resulting in the leakage of blood components. This leads to the release of pro-inflammatory cytokines into the spinal cord. When these inflammatory mediators reach the gastrointestinal tract via systemic circulation, they reduce the thickness of the intestinal mucosa,^20^ disrupt tight junctions,^21^ and impair the intestinal barrier, ultimately contributing to gut microbiota dysbiosis.^22^ Studies have shown that in the acute phase of SCI, gut microbiota undergoes the most significant changes due to decreased intestinal motility and the use of antibiotics, which are mainly characterized by an increase in the quantities of *Firmicutes* and *Proteobacteria*, along with a relative decrease in the quantity of *Bacteroidetes*.^23^ Additionally, the 16S rRNA gene sequencing of fecal samples from 100 patients with SCI revealed that, at the family level, *Prevotellaceae*, *Clostridiaceae*, and *Ruminococcaceae* were reduced to varying degrees. In contrast, the abundance of *Lactobacillaceae*, *Enterobacteriaceae*, and *Verrucomicrobiaceae* increased. At the genus level, the abundance of *Faecalibacterium* and *Coprococcus* decreased, whereas *Streptococcus*, *Enterococcus*, and *Klebsiella* showed varying degrees of increase.^23^ Notably, *Ruminococcaceae* produce short-chain fatty acids (SCFAs), which possess anti-inflammatory properties; thus, their reduction after SCI results in decreased anti-inflammatory metabolites. *Enterococcus* is associated with the increased production of lipopolysaccharides (LPS), which activate the TLR4/MyD88 signaling pathway, leading to the overexpression of pro-inflammatory cytokines. In the context of SCI, such excessive inflammation promotes neuronal death and exacerbates spinal cord damage.^24^ In patients with chronic SCI, the hypodynamic state of the intestine caused by long-term bed rest, along with the continuous release of chronic inflammatory factors such as Interleukin-6 (IL-6) and tumor necrosis factor-α (TNF-α), inhibits the colonization of probiotics. This makes the early gut microbiota disorder tend to be in a steady state and difficult to recover.^25^ Therefore, gut microbiota changes occur after SCI, and these changes can affect the occurrence and development of the disease.

### Spinal cord injury and cognitive dysfunction

Cognitive impairment refers to a decline or disruption in cognitive functions caused by various factors, primarily manifested as reduced abilities in thinking, memory, language comprehension, and judgment. Studies have found that patients with SCI exhibit gray matter atrophy in specific left hemisphere brain regions, including the middle frontal gyrus, pars triangularis of the inferior frontal gyrus, and the precentral gyrus, as well as the right pars triangularis. Reduction in gray matter volume in these regions correlates with impairments in motor function, memory, and language comprehension.^26^ Moreover, SCI has been shown to reduce neural activity in localized brain areas such as the right angular gyrus of the inferior parietal lobule and the left orbital middle frontal gyrus.^27^ The right angular gyrus is primarily responsible for processing and integrating visual information, while the left orbital middle frontal gyrus is involved in emotion regulation. Therefore, dysfunction in these regions may contribute to visual perception deficits and emotional dysregulation.^28^^,^^29^ Craig et al.^4^ compared 150 patients with SCI with 45 healthy adults and found significantly lower cognitive performance among the SCI group. A systematic review by Sachdeva et al.^30^ analyzed 70 recent studies on cognitive deficits in patients with SCI, of which 38 reported impairments in one or more cognitive domains. The prevalence of cognitive impairment among patients with SCI reached as high as 64%, indicating a significantly elevated risk of developing cognitive dysfunction in this population. Therefore, a close relationship exists between SCI and cognitive impairment. Understanding the interaction and underlying mechanisms between the two is essential for developing effective intervention strategies.

### Gut microbiota and cognitive dysfunction

Dysbiosis of the gut microbiota in the acute phase can compromise intestinal barrier function, leading to increased intestinal permeability. As a result, bacterial metabolites and toxins that are normally restricted from entering the body may cross the intestinal wall into the bloodstream, triggering systemic inflammation. These inflammatory mediators can subsequently reach the brain via circulation, inducing neuroinflammation, damaging neurons, and ultimately resulting in cognitive impairment.^31^ If gut microbiota dysbiosis in the acute phase is not alleviated, it will be amplified and prolonged into chronic gut microbiota dysbiosis. Chronic gut microbiota dysbiosis primarily leads to progressive cognitive decline through the accumulation of microbial metabolites.^32^ Therefore, intervening in early gut microbiota dysbiosis is the key to preventing the occurrence of cognitive impairment. Wu et al.^6^ found that SCI in mice activates asparagine endopeptidase, promoting amyloid plaque formation and hyperphosphorylation of Tau protein, thereby inducing cognitive deficits. This pathological process resembles mechanisms observed in patients with AD. Analysis of fecal samples from 25 patients with AD and 25 healthy controls revealed significant differences in microbial diversity. At the phylum level, patients with AD exhibited reduced abundance of *Firmicutes* and *Actinobacteria*, but increased levels of *Proteobacteria* and *Bacteroidetes*. At the family level, there was a decrease in *Clostridiaceae*, *Peptostreptococcaceae*, and *Turicibacteraceae*, alongside an increase in *Porphyromonadaceae* and *Rikenellaceae*.^33^ *Lactobacillus* plantarum is a member of the *Firmicutes*. Studies have shown that the colonization of *Lactobacillus* plantarum can improve cognitive function in aged mice.^34^ Additionally, the pathogenesis of cognitive impairment following SCI shares similarities with PD, primarily involving the dysfunction of dopaminergic neurons in the central nervous system, leading to spatial memory deficits.^35^^,^^36^ In a comparative analysis of fecal samples from 65 patients with PD and 38 healthy individuals, a reduction in *Firmicutes* and an increase in *Proteobacteria* and *Bacteroidetes* were observed at the phylum level. At the family level, *Clostridiaceae, Prevotellaceae*, and *Enterococcaceae* were reduced, while *Bifidobacteriaceae* and *Enterobacteriaceae* were elevated.^37^ At the genus level, *Enterococcus* and *Shigella* were increased, *Faecalibacterium* and *Haemophilus* were reduced.^38^ Moreover, the colonization of *Faecalibacterium* can alleviate the symptoms of PD.^39^ Similarly, patients with severe depression also exhibit cognitive deficits associated with dopaminergic dysregulation. 16S rRNA sequencing has shown that in depressed patients with cognitive impairment, the phylum *Firmicutes* is decreased, while *Bacteroidetes*, *Proteobacteria*, and *Actinobacteria* are increased. At the family level, *Clostridiaceae* and *Peptostreptococcaceae* are reduced, whereas *Oscillospiraceae* is elevated.^40^ A Mendelian randomization analysis indicated that a reduction in *Clostridiaceae* may exacerbate cognitive dysfunction,^41^ while the colonization of *Clostridium* can alleviate depression-like behaviors in mice.^42^Notably, a similar decline in *Clostridiaceae* abundance has been observed in patients with SCI,^23^ consistent with trends seen in AD, PD, and severe depression. These findings suggest that gut microbiota undergo significant alterations following SCI and that these changes are closely associated with cognitive impairment.

Collectively, these observations highlight the strong association between gut microbiota dysbiosis and both SCI and cognitive impairment. However, the mechanistic pathways linking post-SCI microbiota alterations to cognitive dysfunction remain inadequately explored. Therefore, further investigation into this relationship is critical for understanding disease progression and developing targeted therapeutic strategies.

## Mechanisms by which gut microbiota influences cognitive dysfunction after spinal cord injury

**Figure 2:**
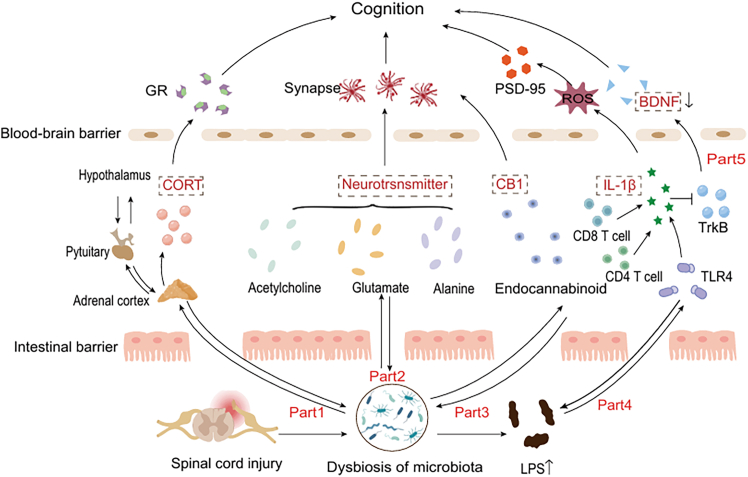
The specific mechanisms by which the gut microbiota affects cognitive function After spinal cord injury, the gut microbiota changes, the adrenal function is impaired, and cortisol levels are altered. Cortisol can bind to glucocorticoid receptors in brain regions, affecting neuronal function and, in turn, cognitive function. Second, gut microbiota dysbiosis modifies the endogenous cannabinoid system in the gut, altering the synthesis and degradation of cannabinoid receptor type 1, disrupting synaptic plasticity, and influencing cognitive function. In addition, there is a close connection between the gut microbiota and the nervous system, which can release various neurotransmitters such as acetylcholine, glutamate, and alanine. These neurotransmitters affect the excitability of synapses and thus influence cognitive function. After spinal cord injury, gut microbiota dysbiosis occurs, and the increased lipopolysaccharides (LPS) affect the secretion of interleukin-1β (IL-1β) through the TLR4/MyD88 signaling pathway, thereby generating excessive reactive oxygen species (ROS). ROS will inhibit the expression of postsynaptic density protein −95, affecting synaptic plasticity and, consequently, cognitive function. Moreover, the increase in IL-1β leads to a decrease in brain-derived neurotrophic factor (BDNF) by inhibiting the TrkB signaling pathway, and the reduction of BDNF in the brain also affects cognitive function.

Cognitive impairment following SCI negatively impacts patient prognosis. Studies have shown that gut microbiota imbalance occurs after SCI and that this dysbiosis is closely related to cognitive function.^43^^,^^44^ With the continuous advancement of modern scientific research technologies, we have gradually recognized that the gut microbiota influences the occurrence and development of cognitive function primarily through the following pathways (Figure 2), which have been verified in preclinical and clinical experiments (Table 1).

**Table 1:** The mechanisms by which gut microbiota affects cognitive function

Preclinical/clinical trials	Gut microbiota	Mechanism	Cognitive function	Reference
Male piglets/Patients with Cushing’s syndrome	*Ruminococcu*↓	CORT↓	↓	Zeng et al., Pivonello et al., Zhang et al., Kruse et al.^45^^,^^46^^,^^47^^,^^48^
Zebrafish with anxiety-like behaviors/Elderly patients	*Lactobacillus*↑/*Prevotella*↑	Glutamate↑/Alanine↑	↑	Ostfeld et al., Lohani et al., Chen et al., Mhanna et al., Xue et al., Iwata et al.^49^^,^^50^^,^^51^^,^^52^^,^^53^^,^^54^
Diabetic rats/patients with AD	*Bifidobacteria*↑ *Lactobacilli*↑	CB1↑	↑	Chen et al., Hussein et al., Morsli et al.^55^^,^^56^^,^^57^
Rats/Patients with severe pulmonary tuberculosis	*Klebsiella*↑/*Faecalibacterium*↓、*Veillonella*↓	IL-1β↑	↓	Yu et al., Lee et al., Fu et al., Chai et al., Lu et al.^58^^,^^59^^,^^60^^,^^61^^,^^62^
Elderly mice/patients with AD	*Prevotella*↑	BDNF↑	↑	Carlos et al., Yun et al., Petrosyan et al.^63^^,^^64^^,^^65^

### Cortisol

Cortisol (CORT) is a glucocorticoid hormone secreted by the adrenal cortex under the regulation of the HPA axis. It plays a critical role in glucose, protein, and fat metabolism, as well as stress responses.^55^ Studies have shown that CORT affects neuronal function by binding to glucocorticoid receptors (GRs) in the hippocampus, amygdala, and prefrontal cortex, thereby influencing cognition.^66^ After SCI, the neural connection between the hypothalamus and spinal cord is disrupted, leading to adrenal dysfunction and increased CORT production. This results in reduced upstream HPA hormones, such as adrenocorticotropic hormone (ACTH), due to negative feedback, thereby disrupting HPA axis homeostasis.^45^ Moreover, the gut microbiota also plays an essential role in maintaining HPA axis homeostasis.^67^ Mudd et al.^68^ using 16S rRNA gene sequencing on 24 male piglets, found that elevated levels of *Ruminococcus* lowered CORT levels, influencing HPA axis activity. A clinical trial study showed that high levels of CORT in the body can induce Cushing's syndrome and cause cognitive impairment.^46^^,^^47^ In addition, the concentration of *Ruminococcus* also increases after SCI.^23^ Therefore, the elevated concentration of *Ruminococcus* after SCI may reduce CORT levels by affecting the HPA axis, thereby impairing neuronal function and subsequently contributing to the development of cognitive dysfunction.

### Neurotransmitters

Neurotransmitters are chemical messengers that transmit signals between neurons, enabling the nervous system to coordinate and regulate physiological and behavioral functions. Gut microbiota can release various neurotransmitters, such as acetylcholine, glutamate, and alanine, which play key regulatory roles in the brain, modulating cognition, emotion, attention, learning, and memory.^48^^,^^49^^,^^50^

#### Acetylcholine

Acetylcholine plays a vital role in regulating neurogenesis, neuronal differentiation, synaptic plasticity, and neuroprotection in the central nervous system. The hippocampus, which is rich in nicotinic acetylcholine receptors, receives acetylcholine from cholinergic neurons, allowing the excitation and integration of sensory and memory-related information across brain regions, thereby affecting cognitive function.^51^ Studies have shown that damage to cholinergic receptors in the hippocampal CA3 region interrupts neural signaling and causes cognitive impairment.^69^ Sun et al.^70^ found that administering M1 cholinergic receptor agonists to the prefrontal cortex of AD mice improved performance in object recognition and Morris water maze tests, effectively enhancing cognitive function. Since SCI leads to reduced acetylcholine levels,^71^ a close relationship exists between acetylcholine and cognition. 16S rRNA gene sequencing showed that decreased *Clostridium* and increased *Streptococcus* abundance could impair normal acetylcholine function in patients with myasthenia gravis,^72^ indicating that gut dysbiosis can impair acetylcholine function. Similar trends in gut microbiota are observed in patients with SCI. Therefore, reduced *Clostridium* and increased *Streptococcus* after SCI may impair acetylcholine function and contribute to cognitive dysfunction.

#### Glutamate

Glutamate is one of the primary excitatory neurotransmitters in the CNS and plays a key role in synaptic transmission. By enhancing excitatory signaling, glutamate facilitates neuronal communication and supports cognitive function.^73^ However, excessive glutamate can lead to calcium influx into postsynaptic neurons, causing excitotoxicity and neuronal damage, ultimately resulting in cognitive impairment.^74^ Thus, both high and low glutamate levels can be detrimental. SCI has been shown to increase glutamate concentration, causing neurotoxicity and cognitive dysfunction,^75^A metagenomic association study revealed that the abundance of *Bacteroides* was significantly reduced in obese individuals and was negatively correlated with serum glutamate levels.^76^ Therefore, reduced *Bacteroides* may be linked to elevated glutamate and excitotoxicity, contributing to cognitive impairment. Moreover, 15% of *Lactobacilli* can produce glutamate,^77^ supplementation with *Lactobacilli* can alleviate anxiety-like behaviors in zebrafish and improve their cognitive function.^78^ Meanwhile, the content of *Lactobacilli* tends to decrease after SCI.^23^Therefore, the concentration of glutamic acid may also decrease after SCI, and a reduction in glutamic acid levels can lead to cognitive impairment.^52^ In summary, the decreased levels of *Bacteroides* and *Lactobacillus* after SCI may impair the normal function of glutamate, thereby disrupting the transmission of neural excitation and ultimately contributing to the development of cognitive dysfunction.

#### Alanine

Alanine is involved in nitrogen transport from muscle to the liver, where nitrogen is converted into urea and excreted, maintaining nitrogen balance. Proper nitrogen balance is essential for brain protein synthesis and neuronal function. Disruption can impair neurotransmitter, neuropeptide, and receptor protein synthesis, affecting cognition.^79^ A double-blind, randomized study of 100 elderly individuals showed that β-alanine supplementation improved cognitive function.^80^ Metagenomic analysis in ruminants revealed that *Prevotella* abundance positively correlated with amino acids, carboxylic acids, and fatty acids, mainly affecting alanine levels.^53^ In addition, animal studies have found that some *Enterobacter* and *Klebsiella* in mice can produce D-alanine.^54^ Therefore, changes in intestinal flora can affect changes in alanine content. 16S rRNA sequencing in patients with SCI revealed decreased *Prevotella* and increased *Enterobacter* and *Klebsiella* abundance.^23^^,^^81^ Therefore, the alterations in *Prevotella*, *Enterobacter*, and *Klebsiella* after SCI may affect alanine levels, thereby interfering with protein synthesis and subsequently impacting cognitive function.

Therefore, SCI-induced gut microbiota imbalance alters microbial metabolites, potentially affecting neurotransmitter levels (acetylcholine, glutamate, and alanine) and contributing to cognitive dysfunction.

### Cannabinoid type 1 receptor

Cannabinoid type 1 receptor (CB1) is a primary receptor in the central nervous system through which the endocannabinoid system exerts its effects. When endogenous cannabinoids, such as anandamide and 2-arachidonoylglycerol,^82^ bind to high-affinity G protein-coupled receptors such as CB1R and Cannabinoid receptor 2 (CB2), they act retrogradely—being released from postsynaptic neurons and traveling backward across the synapse to temporarily inhibit presynaptic release of inhibitory GABA or excitatory glutamate, thereby influencing cognitive function.^44^ Gut microbiota dysbiosis significantly alters the expression of CB1, CB2, and the enzymes involved in endocannabinoid synthesis and degradation in the gut.^83^ Studies have shown that the administration of probiotics containing *Bifidobacterium* and *Lactobacillus* in patients with AD can upregulate CB1 and CB2 expression, improving cognitive function.^84^ Similarly, an animal study confirmed that increasing levels of *Bifidobacterium* and *Lactobacillus* could enhance CB1 expression and improve cognitive function in diabetic rats.^56^ After SCI, the abundance of *Bifidobacterium* and *Lactobacillus* decreases,^57^ a trend similar to that seen in AD and diabetes. Therefore, the reduced abundance of *Bifidobacterium* and *Lactobacillus* after SCI may affect the release of inhibitory GABA or excitatory glutamate from presynaptic terminals by influencing the expression of CB1, thereby contributing to the development of cognitive dysfunction.

### Interleukin-1β

Interleukin-1β (IL-1β) is a key pro-inflammatory cytokine. In the acute phase of SCI, monocyte-macrophages release inflammatory factors such as tumor necrosis factor (TNF-α), IL-1β, and interferon-γ (IFN-γ). These inflammatory mediators cause axonal damage or death, disrupting neural signaling.^85^ High levels of IL-1β impair hippocampal long-term potentiation (LTP),^86^ inhibit glutamate release^87^ reduce calcium influx in hippocampal synaptosomes,^88^ and lead to memory impairment.^89^ Chen et al.^90^ reported that TNF-α and IL-1β stimulate neurons and glial cells to produce reactive oxygen species, which suppress postsynaptic density protein-95 expression and reduce dendritic spine number and size, affecting synaptic plasticity and cognition. Animal studies have found that increased *Klebsiella* correlates with elevated LPS,^58^ which activates the TLR4/MyD88/NF-κB pathway to promote IL-1β release.^59^^,^^60^ In the treatment of patients with tuberculosis with immune checkpoint inhibitors, it was found that patients with lower concentrations of *Faecalibacterium* and *Veillonella* had reduced numbers of peripheral CD4^+^ T cells and CD8^+^ T cells, which affected the secretion of IL-1β.^61^ After SCI, *Klebsiella* levels rise while *Faecalibacterium* and *Veillonella* decrease.^62^ Therefore, increased *Klebsiella* and reduced *Faecalibacterium* and *Veillonella* may disrupt IL-1β regulation and contribute to cognitive dysfunction.

### Brain-derived neurotrophic factor

Brain-derived neurotrophic factor (BDNF) is a neurotrophin widely expressed in the brain, crucial for neuronal growth, differentiation, survival, and synaptic plasticity. It plays key roles in emotion regulation, learning, memory, and cognitive function. Gao et al.^91^ found that BDNF deletion worsens tau phosphorylation and Aβ accumulation, aggravating AD. In addition, IL-1β downregulates the transcription of the BDNF gene by inhibiting TrkB receptor signaling, resulting in synaptic loss and cognitive decline.^63^^,^^92^ Herbal extracts such as resveratrol and curcumin can enhance hippocampal BDNF expression and improve cognitive dysfunction.^93^^,^^94^ Li et al.^95^ quantified BDNF in the cerebellum and spinal cord of SCI mice and found significantly reduced expression, which was restored following fecal microbiota transplantation.^96^ Pourkhodada et al.^97^ also confirmed decreased BDNF in spinal tissue post-SCI. Butyrate is considered a candidate molecule linking gut microbiota to BDNF. An animal study showed that butyrate promotes BDNF mRNA expression in the prefrontal cortex by inhibiting histone deacetylase.^98^ Clinical investigations have shown that *Prevotella* can increase the level of BDNF in the human body, thereby improving cognitive function in patients with AD.^99^ Animal studies have also indicated that *Prevotella* can elevate BDNF levels in the hippocampus and alleviate the occurrence of cognitive impairment through the gut-brain axis.^64^ Therefore, reduced *Prevotella* after SCI^23^ may affect neuronal differentiation and synaptic plasticity by influencing the expression of BDNF, thereby impacting the occurrence and progression of cognitive impairment.

Based on the above, gut microbiota may influence cognitive impairment after SCI through one or more of these five signaling pathways, which are likely interrelated.

## Therapeutic strategies targeting gut microbiota for post-spinal cord injury cognitive dysfunction

Currently, the therapeutic approaches for cognitive impairment after SCI mainly include pharmacological and non-pharmacological treatments. Non-pharmacological treatments further encompass rehabilitation therapy, exercise therapy, neuromodulation, and psychological therapy (Table 2). Pharmacological treatments primarily involve neurotrophic drugs, anti-inflammatory agents, drugs for improving cerebral circulation, and neurotransmitter-regulating drugs. These medications enhance cognitive function post-SCI by increasing acetylcholine release, inhibiting neuroinflammation, and dilating cerebral blood vessels. However, in addition to inducing drug resistance and allergic reactions, long-term administration of these drugs may also cause certain damage to the liver and kidneys.^65^^,^^100^^,^^101^^,^^102^ Rehabilitation therapy mainly consists of targeted training, such as memory training, attention training, and executive function training.^103^^,^^104^ Exercise therapy includes aerobic exercise and balance-coordination training: aerobic exercise can promote the secretion of BDNF, enhance hippocampal neuroplasticity, and improve memory function; balance-coordination training indirectly enhances the brain’s ability to regulate the body through core muscle group exercise and posture control training, thereby facilitating the impact of proprioceptive input on cognition. Nevertheless, long-term rehabilitation and exercise therapy may not only increase the economic burden on patients but also bring about emotional stress.^105^^,^^106^ Neuromodulation mainly involves transcranial magnetic stimulation (TMS), transcranial direct current stimulation (tDCS), and spinal cord electrical stimulation (SCS). These stimulation methods improve cognition by increasing cortical excitability and remodeling neural network connections. However, this therapeutic approach may carry risks such as headaches and burns.^107^^,^^108^ Meanwhile, speech and psychological therapy may involve personal privacy and sensitive information, posing a risk of privacy leakage.^109^ Therefore, there is an urgent need to explore new therapeutic strategies to alleviate cognitive impairment after SCI. As previously discussed, gut microbiota influences cognitive dysfunction following SCI through various mechanisms. Thus, regulating and improving the gut microbial community—known as gut microbiota-based therapy—may provide a novel approach for the prevention and treatment of post-SCI cognitive impairment. Such approaches include probiotic and prebiotic supplementation, fecal microbiota transplantation (FMT), and dietary interventions.

**Table 2:** Current approaches to treating cognitive dysfunction after SCI

therapeutic approaches		Reference
pharmacological treatments	neurotrophic drugs, anti-inflammatory drugs, drugs for improving cerebral circulation, neurotransmitter-regulating drugs	Ji et al., Qiao et al., Yang et al., Zhang et al.^100^^,^^101^^,^^102^^,^^103^
rehabilitation therapy	memory training, attention training, executive function training	Baylo-Marín et al., Blume et al.^104^^,^^105^
exercise therapy	aerobic exercise, balance and coordination training	Zwijgers et al., Wenqiang et al.^106^^,^^107^
Neuromodulation therapy	TMS, tDCS, SCS	Kumru et al., Wallace et al.^108^^,^^109^
psychological therapy	verbal alleviation of depression and anxiety, behavioral alleviation of depression and anxiety	Kelly et al.^110^

Probiotics can effectively regulate the intestinal environment, restore microbial balance, and maintain physiological homeostasis, thereby influencing the development and progression of cognitive function. They represent a safe and reliable treatment option. Probiotics may modulate cognitive function by altering HPA axis activity and regulating cortisol release.^110^ Additionally, SCFAs produced by gut bacteria after probiotic supplementation can enhance LTP and memory formation by increasing histone acetylation.^111^ Probiotics containing *Lactobacillus* and *Bifidobacterium* strains have also been shown to enhance the activity of regulatory T cells and increase the secretion of neuroactive metabolites and neurotransmitters, thus improving central nervous system disorders.^112^ In addition, probiotics can reduce the entry of intestinal inflammatory factor IL-1β into the bloodstream, significantly increase serum BDNF, and ameliorate cognitive impairment.^113^ However, most of the existing relevant studies are animal experiments with insufficient levels of clinical evidence. Furthermore, there are significant differences in metabolite profiles and intestinal colonization abilities among different probiotic strains. In the future, it is necessary to clarify the optimal intervention regimens and applicable populations of probiotics through mechanistic studies and standardized clinical trials, so as to promote their precise application in the cognitive rehabilitation of SCI.

FMT is an emerging method in which microbiota from the feces of a healthy donor are transplanted into a recipient’s gut, aiming to restore microbial balance and reestablish intestinal homeostasis. Studies have shown that transplanting gut microbiota from healthy mice into mice with SCI can improve motor recovery, promote neuronal survival and axonal regeneration, and ultimately enhance cognitive function.^22^ An SCI patient with *Clostridium* difficile infection experienced resolution of the infection after receiving FMT from his healthy son, with no recurrence observed during the 12-week follow-up.^114^ Patients with PD with cognitive dysfunction, such as those with SCI, experienced alleviated symptoms after FMT. Moreover, no adverse reactions occurred during the entire treatment process and subsequent follow-up observations. This fully demonstrates the safety and efficacy of FMT in clinical applications, bringing new hope for the treatment of such patients.^115^ However, the clinical application of FMT faces significant challenges. Due to individual variations, patients with SCI have compromised immune function, making them susceptible to various bacterial infections, thus requiring more rigorous clinical trial procedures. Additionally, FMT involves ethical issues, and full informed consent from both donors and patients should be obtained prior to clinical research.^116^

Dietary regulation mainly focuses on increasing the intake of dietary fiber. Foods such as fruits, vegetables, grains, coffee, and tea can alleviate oxidative stress and inflammatory responses associated with SCI.^117^ Moreover, resveratrol—a polyphenol found in red wine—has been shown to support the recovery of neurological function after SCI by reshaping the gut microbiota and increasing butyrate levels.^118^ The ketogenic diet is a dietary pattern characterized by high fat, low carbohydrate, and moderate protein. It can increase the level of β-hydroxybutyrate in the brain of patients with SCI, promote the expression of synaptic proteins, enhance motor function in patients with SCI, alleviate pain and neuroinflammation, and reduce dysfunction and mortality.^119^ In addition, ketosis can selectively inhibit certain *Bifidobacterium* strains associated with the induction of pro-inflammatory Th17 cells, thereby conferring anti-inflammatory effects to improve SCI.^120^ Therefore, dietary regulation is more economical and convenient than drug therapy, and the combination of dietary intervention and microbiota-targeted therapy has become an auxiliary approach for cognitive rehabilitation in SCI.

Therefore, gut microbiota-based therapies—including probiotic and prebiotic supplementation, FMT, and dietary interventions—offer protective effects for both spinal cord injury and associated cognitive dysfunction.

## Limitations of the study

Although a preliminary understanding has been gained regarding the impact of gut microbiota on cognitive function following SCI, numerous limitations still exist. From the perspective of research models, animal experiments, while serving as a crucial research tool, exhibit discrepancies from human physiological and pathological conditions. Commonly used animal models of SCI fail to fully replicate the complexity of human SCI. For instance, human SCI is often caused by multiple complex factors such as car accidents and falls from heights, resulting in diverse injury severities and types. In contrast, animal models typically involve the induction of a single type of injury under relatively controlled experimental conditions. Additionally, the experimental cycle of animal models is relatively short, making it difficult to adequately reflect the long-term pathophysiological processes and cognitive function changes in humans after SCI. Furthermore, variations in the baseline composition of gut microbiota among different animal strains may interfere with the research findings on the relationship between gut microbiota and cognitive impairment post-SCI, thereby affecting the generalizability and extrapolation of the research results. From the perspective of research methods, certain limitations are inherent in the detection techniques for gut microbiota. Although 16S rRNA gene amplicon sequencing is widely used, it can only identify bacteria at the genus level, not the species level. Moreover, it may miss the detection of some low-abundance bacteria, making it challenging to fully reflect the true composition of the gut microbiota. Metagenomic sequencing, while capable of providing more detailed genetic information of the microbiota, is limited in its large-scale application due to its high cost and complex data analysis. When detecting gut microbiota metabolites, metabolomics technology also faces issues such as limited detection sensitivity and difficulty in accurately qualitative and quantitative analysis of some metabolites, which hinders the in-depth exploration of the mechanisms underlying the role of gut microbiota metabolic pathways and related metabolites in cognitive impairment after SCI. In terms of mechanism research, although it has been proposed that gut microbiota affects cognitive function after SCI through pathways involving cortisol, neurotransmitters, CB1, IL-1β, and BDNF, the interrelationships and synergistic effects among these pathways remain unclear. In terms of clinical research, deficiencies are also evident in clinical studies. On one hand, patients with SCI exhibit significant individual differences, including variations in injury cause, injury site, injury severity, and baseline health status, which makes it difficult to unify and summarize research results. On the other hand, clinical trials investigating gut microbiota modulation for the treatment of cognitive impairment post-SCI generally have small sample sizes, short study durations, and lack long-term follow-up data, making it challenging to evaluate the long-term efficacy and safety of the treatments. Furthermore, the intervention measures in existing clinical studies are diverse—for example, there is a lack of standardized protocols for donor selection, transplantation methods, and frequency in FMT, as well as for the type, dosage, and course of probiotics. This leads to the poor comparability of research results and makes it difficult to draw definitive clinical recommendations.

## Conclusion and outlook

After SCI, patients not only face physical disabilities but also cognitive impairments, which severely affect their quality of life and rehabilitation progress. This article discusses the close relationship between gut microbiota and cognitive dysfunction after SCI. SCI can disrupt the gut barrier, leading to dysbiosis, manifested by changes in the proportions of *Firmicutes*, *Bacteroidetes*, and *Verrucomicrobia*, as well as alterations in the abundance of *Ruminococcaceae*, *Clostridiaceae*, and other gut bacteria. These changes in gut microbiota can impact the central nervous system through the regulation of cortisol, IL-1β, cannabinoid receptor 1, neurotransmitter systems, and brain-derived neurotrophic factor, ultimately leading to cognitive dysfunction.

Although there is a preliminary understanding of the influence of gut microbiota on cognitive function, many challenges and unknowns remain in this field. The gut microbiota is a complex microbial community, and understanding the specific impact of each microorganism on cognitive function and its interactions is highly intricate. Furthermore, there is a need for a deeper understanding of the precise relationship between gut microbiota and the central nervous system, as well as how dysbiosis occurs and is transmitted. Additionally, individual differences in gut microbiota composition may result in varying effects on cognitive function after SCI, so clinical studies require more diverse and larger sample sizes to validate the relationship between gut microbiota and cognitive dysfunction post-SCI, providing more reliable evidence for clinical treatment. Therefore, in future research, we can expect in-depth studies in molecular biology, neuroscience, and microbiology to uncover the precise mechanisms by which gut microbiota contributes to cognitive dysfunction after SCI, discover new therapeutic strategies, and provide new approaches and treatments for the rehabilitation of patients with SCI.

## Acknowledgments

This work was supported by the 10.13039/501100001809National Natural Science Foundation of China (82560366 to Xiaowei Chang), 10.13039/501100001809National Natural Science Foundation of China (82560280 to Yanling Yang), Graduate Education Innovation Program of Yan’an University (YCX2024102 to Chunping Tian), Key Industry Chain Project of Yan'an Science and Technology Bureau (2024SLZDCY-021 to Xiaowei Chang), Yan'an University Doctoral Research Launch Project (Xiaowei Chang), and the General Project of Shaanxi Provincial Department of Science and Technology (2024SF-YBXM-037 to Yanling Yang).

## Author contributions

C.T. writing and revising the text. J.W. and L.X. collecting data. J,D. and Q.H. supervision. X.C. and Y.Y. conceptualizing and guiding the article. All authors give final approval of the version to be published and give an agreement to be accountable for all aspects of the work in ensuring that questions related to the accuracy or integrity of any part of the work are appropriately investigated and resolved.

## Declaration of interests

The authors declare no conflicts of interest.
